# When Two Maladies Meet: Disease Burden and Pathophysiology of Stroke in Cancer

**DOI:** 10.3390/ijms232415769

**Published:** 2022-12-12

**Authors:** Ming-Yee Sun, Sonu M. M. Bhaskar

**Affiliations:** 1 Global Health Neurology Lab, Sydney, NSW 2000, Australia; 2 Neurovascular Imaging Laboratory, Clinical Sciences Stream, Ingham Institute for Applied Medical Research, Liverpool, NSW 2170, Australia; 3 UNSW Medicine and Health, University of New South Wales (UNSW), South Western Sydney Clinical Campuses, Sydney, NSW 2170, Australia; 4 Department of Neurology & Neurophysiology, Liverpool Hospital and South West Sydney Local Health District (SWSLHD), Liverpool, NSW 2170, Australia; 5 NSW Brain Clot Bank, NSW Health Pathology, Sydney, NSW 2170, Australia; 6 Stroke & Neurology Research Group, Ingham Institute for Applied Medical Research, Liverpool, NSW 2170, Australia

**Keywords:** stroke, cancer, thrombosis, blood clot, chemotherapy, radiotherapy

## Abstract

Stroke and cancer are disabling diseases with an enormous global burden, disproportionately affecting vulnerable populations and low- and middle-income countries. Both these diseases share common risk factors, which warrant concerted attention toward reshaping population health approaches and the conducting of fundamental studies. In this article, an overview of epidemiological trends in the prevalence and burden of cancer and stroke, underlying biological mechanisms and clinical risk factors, and various tools available for risk prediction and prognosis are provided. Finally, future recommendations for research and existing gaps in our understanding of pathophysiology. Further research must investigate the causes that predispose patients to an increased risk of stroke and/or cancer, as well as biomarkers that can be used to predict growing morbidity and mortality.

## 1. Introduction

Stroke is a debilitating disease with a significant morbidity burden on individuals and ever-increasing costs imposed on public health systems worldwide [1]. It stands as the second leading cause of disability-adjusted life years (DALYs) globally, with a disproportionate number of stroke-related deaths and DALYs precipitating in low- and middle-income countries (LMICs) [2]. Within Australia, a 2020 report released by Deloitte found the economic impact of stroke to be 6.2 billion Australian dollars in direct financial costs, with a further 26 billion dollars attributable to premature mortality and DALYs [3]. A similarly dire picture exists for cancer, which accounts for almost 10 million deaths worldwide each year [4]. Estimates by the International Agency for Research on Cancer (IARC) suggest that the total cost of cancer amounts to USD 1.16 trillion annually, with LMICs again disproportionately impacted, despite having lower rates of cancer, as a majority of deaths are accounted for in socioeconomically disadvantaged areas [5]. Given this substantial burden posed by stroke and cancer individually, and the various shared risk factors in the pathophysiology of cancer and stroke, such as dyslipidemia, diabetes mellitus, and hypertension, the heightened stroke risk in cancer patients, especially those undergoing radiation therapy [6], these two diseases become an even more pertinent issue to address [7]. In this article, we provide a comprehensive update on the burden of stroke in cancer, the pathophysiology of stroke in cancer, as well as perspectives on the risk of stroke in cancer patients undergoing chemotherapy, radiation, or both therapies.

## 2. The Burden of Stroke and Cancer

Table 1 provides an overall assessment of burden, prevalence, DALY, and financial burden in stroke, cancer, and cancer-associated stroke. Much needs to be accomplished to reduce the global financial burden of cancer and stroke and the life-changing impact it has on the patients themselves. Of particular concern is the inordinate burden that LMICs face, where despite having lower rates of cancer, a majority of deaths are accounted for in these socioeconomically disadvantaged areas. A similar parallel is drawn in the context of stroke, whereby an unfair 87% of DALYs are lost within LMICs [8]. More robust education, screening, availability, and accessibility of treatment must all be addressed in order to reduce these gaps.

### 2.1. Burden of Stroke

Stroke afflicts millions of individuals worldwide, with estimates from the 2019 GBD study estimating 12.2 million incidents and 101 million prevalent cases [2]. Ischaemic stroke accounted for 62.4% of incidences, 27.9% by intracerebral hemorrhages, and 9.7% by subarachnoid hemorrhages. Furthermore, 6.55 million deaths occurred due to stroke. Though rates of stroke incidence, prevalence, mortality, and DALY rates declined in the last few decades, the absolute number of individuals developing stroke, dying, or becoming disabled from stroke doubled. This is cause for further concern given that most of the stroke burden occurs in LMICs, which shoulder 80% of incident strokes, 87% of deaths, and 89% of all stroke-related DALYs [24]. Currently, inadequate gaps in the care of stroke in LMICs include longer waiting times in the emergency department, a paucity of equipped stroke-dedicated units, funding deficiencies/financial difficulties in purchasing potentially lifesaving trans plasminogen activator (tPA), and under-established secondary prevention public health promotion strategies [25].

Within the context of Australia, in 2018, the Australian Institute of Health and Welfare (AIHW) estimated 387,000 prevalent stroke cases, declining from 1.7% in 2003 to 1.3% in 2018 [10]. Stroke incidence in 2017 came to approximately 38,000, falling 24% from 2001 to 2017. Stroke as the underlying cause of death accounted for 8400 lives lost in 2018-5.3% of all deaths that year. Death rates from stroke also fell by 75% from 1980 to 2018. These all point toward improved prevention, treatment, and post-stroke care within the Australian health system.

Disparities exist within Australia as well, with Aboriginal and Torres Strait Islander peoples experiencing higher rates of stroke in comparison to non-Indigenous Australians, being 1.6 times more likely to be hospitalized for a stroke and disproportionately accounting for 2.3 times the overall burden of disease [26]. Geographical disparities in stroke diagnosis and care are another important factor mediating access and outcomes after stroke [27,28,29,30]. Stroke hospitalization rates are reportedly higher in remote and very remote areas in comparison to major cities, indicating various gaps of insufficient care due to increased travel times, lack of education, and greater limitations in supplies and available treatment [10]. Finally, the burden of stroke is more pronounced across lower socioeconomic areas relative to those from the highest areas.

### 2.2. Burden of Cancer

Cancer remains a leading cause of death worldwide, with the International Agency for Research on Cancer (IARC) estimating 19.3 million new cancer cases worldwide in 2020, alongside 10 million deaths from cancer [11]. The combination of an aging global population with an increasing lifespan, and ever-worsening environmental risk factors thus mean that the burden of cancer will only increase in future years. In Australia, estimates from the AIHW show that in 2021, there were 151,000 new cancer diagnoses, and 49,000 deaths [16]. Furthermore, 9% of the entire health system expenditure is attributed to cancer, with estimates from the 2015–2016 period culminating in an overall AUD 10.1 billion expense, with AUD 9.1 billion spent on diagnosis and treatment, and AUD 409 million on the nationwide bowel, breast, and cervical screening programs [16]. Though cancer mortality and diagnosis rates are trending downward due to vast improvements in screening programs and cancer care, discrepancies exist across the Australian population regarding both cancer diagnosis and mortality. Upon comparing Indigenous and non-Indigenous populations, the former group is 14% more likely to be diagnosed with cancer in their lifetime, and 20% less likely to survive 5 years after diagnosis. This gap continues in terms of the hospital care received, whereby Indigenous people accounted for a higher proportion of public hospital admitted care, at 73% vs. 52%, whilst 33% of non-Indigenous people receive care in private hospitals compared to 6% of Indigenous people [31]. This calls into question the disparity in care received, ability to access cancer specialists, and novel clinical trials—all of which might improve prognosis.

Geographical differences, especially in indigenous populations, also mediate outcomes after cancer [32]. Survival after cancer diagnoses declines with increasing remoteness (55% in very remote areas, 63% in major cities), in part, reflecting the poorer survival rates for Indigenous Australian populations also who reside in remote areas [16]. Incidence of diagnosis for the common cancers of lung cancer and head and neck cancer also increases with remoteness, with breast cancer being the main exception, with cases highest in major cities at 127 cases per 100,000 females vs. 89 cases per 100,000 females in very remote areas [16].

Social determinants of health also impact the entire continuum of cancer care [16]. The relationship between cancer incidence and the socioeconomic area was also detailed, with age-standardized incidence rates tending to increase with one’s disadvantage, consistent for colorectal, lung, head and neck, kidney, pancreatic, liver, cervical, and uterine cancers. However, for breast and prostate cancers, incidence rates decrease with increasing disadvantage. However, regardless of cancer, the 5-year observed cancer survival rate is consistently highest in areas of least disadvantage. From 2012 to 2016, individuals had a 56% 5-year survival rate when combining all cancers, compared to 68% in areas with the least disadvantage. This corresponds with cancer mortality rates, whereby there are 185 in 100,000 deaths in the most socioeconomically disadvantaged areas, vs. 130 per 100,000 in the least disadvantaged [16].

This mismatch is further exacerbated and exposed on a worldwide scale. Across the globe, the interplay between socioeconomic status and cancer risk is also of particular concern (Table 1). The 2019 JAMA Global Burden of Disease (GBD) study revealed that whilst 50% of cancer cases occur in high socio-demographic index (SDI) countries, these countries account for only 30% of cancer deaths, 25% of cancer DALYs, and 23% of cancer years of life lost (YLLs) [17]. This points to the current disparity in the burden experienced between developed and developing countries, whereby countries with limited resources are inadequately equipped in comparison to the rest of the globe with regards to screening programs, immunization, and cancer care, all of which would decrease this burden. The case of cervical cancer most adroitly explores this inequality, whereby country SDI plays a major role, as preventable cancer with a well-established, cost-effective vaccination and screening programs available, incidence rates are falling at slower rates in low SDI countries due to insufficient financial resources to instigate comprehensive screening [33].

The COVID-19 pandemic also caused major disruption to the standard processes of cancer screening, treatment, and care [34,35,36]. The reduction in resources and access to healthcare only served to exacerbate already existing disparities between urban and remote populations, and those who are socioeconomically disadvantaged [36].

## 3. Risk Factors for Stroke in Cancer

Though individual entities, the interactions between cardiovascular disease (CVD) and cancer are complex and suggest a shared pathophysiology, in particular, chronic inflammation [7]. A retrospective analysis of 1274 stroke patients, in which 12% had an additional cancer diagnosis, the researchers identified cerebrovascular risk factors for cancer patients as hypertension, atrial fibrillation, coronary vessel disease, smoking, hypercholesterolemia, and diabetes mellitus, which is in line with those of non-cancer populations.

Conditions such as obesity, hyperglycemia, hypertension, and hypertriglyceridemia, which are linked with CVD, are of particular significance due to their inflammatory effects [37]. As inflammation underpins each stage of atherosclerosis, atherosclerotic emboli are thus implicated in stroke risk within cancer patients, with conditions such as hypertension, smoking, dyslipidemia, and insulin resistance contributing toward the progression of atherosclerosis. Within cancer, the role of inflammation is also clear, with multiple cancer phenotypes triggered by an infection or chronic inflammatory diseases, such as human papillomavirus and cervical cancer, H. pylori and gastric cancer, and Epstein–Barr virus and lymphoma [9].

**Figure 1:**
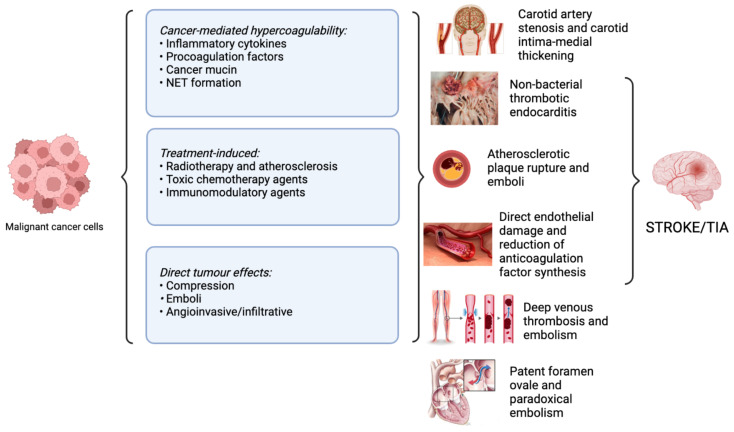
Overview of pathophysiological mechanisms of stroke in cancer patients. Abbreviations: NET: neutrophil extracellular trap; TIA: transient ischemic attack.

Cancer patients also face unique risk factors that contribute toward cerebrovascular events; namely the direct tumor effects, cancer-induced coagulation disorders, and the impact of radiotherapy and chemotherapy must be considered. The multi-faceted avenues by which stroke can occur in cancer patients are illustrated in Figure 1.

## 4. Pathophysiological Mechanisms Underlying Stroke in Cancer

The unique pathogenesis of stroke in cancer patients is multifaceted and can be attributed to multiple aetiological pathways, as summarized in Figure 1.

**Figure 2:**
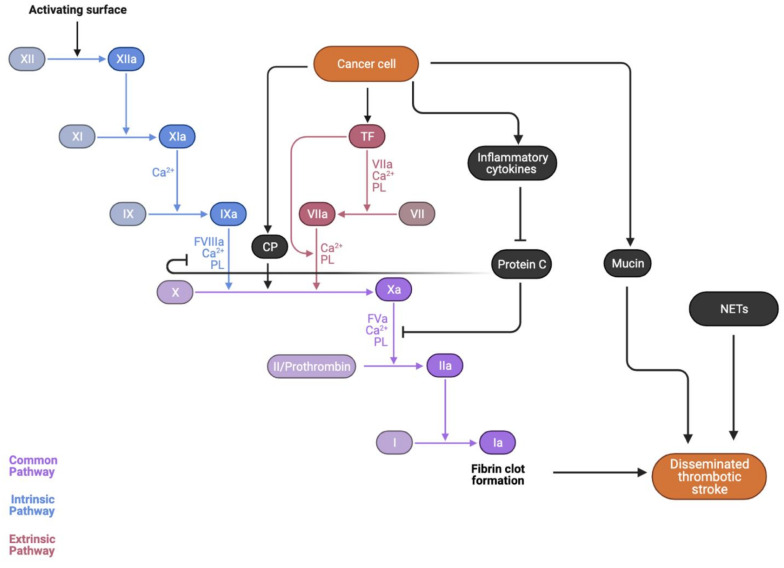
Cancer-induced hypercoagulability on a molecular level. Impact of cancer on the coagulation cascade. Inflammatory cytokines such as TNF-a, IL-1, and IL-6 induce endothelial cells, monocytes, and cancer cells to express tissue factor (TF), exerting a parallel action to potentiate the coagulation cascade. Protein C, which regulates Factor VIIIa and Factor Va (cofactors in the activation of Factor X and prothrombin), is inhibited by these cytokines. TF binds to Factor VII, potentiating the coagulation cascade by proteolytically activating Factor IX and Factor X, thus leading to the formation of thromboses and subsequent stroke. CP, a cysteine protease, is released in most malignancies and directly cleaves. Factor X → Xa independently of FVII, resulting in thrombin generation. Mucin is a potent procoagulant that directly activates prothrombin into thrombin, mostly secreted by adenocarcinomas. Abbreviations: CP: cancer procoagulant; TF: tissue factor; PL: phospholipids; NETs: neutrophil extracellular trap; TNF: tumor necrosis factor; IL: interleukin; and CP: cancer procoagulant.

Direct compressive effects of the tumor itself, tumor emboli, and metastatic angioinvasion are all potentiate the risk of ischaemic or hemorrhagic stroke [38,39]. Furthermore, a characteristic of cancer is the hypercoagulable state induced by the cytokine storm, whereby excessive local release of procoagulants, such as tumor necrosis factor (TNF)-a, interleukin (IL)-1, and IL-6, results in a hypercoagulable state [40]. When exacerbated by the activity of tissue factor (TF), cancer procoagulant (CP), and cancer mucin, as demonstrated in Figure 2, the coagulation cascade is activated and accelerated, potentiating a thromboembolic event [41]. This constant interaction between cytokines and exposed endothelium can lead to a damaged surface primed for platelet aggregation, giving rise to sterile, thrombotic vegetations to deposit on cardiac valves [42]. This development of non-bacterial thrombotic endocarditis (NBTE) further increases cancer patients’ thrombotic and stroke risk [43].

Cancer treatment therapies were also implicated in worsening a patient’s stroke risk, with radiotherapy-induced atherosclerotic acceleration and chemotherapy-induced endothelial damage both associated with increased embolic and stroke risk [38,44]. Of note is the role of radiotherapy in inciting and accelerating the atherosclerotic process within cancer patients and leading to plaque emboli, which ultimately lodge in the cerebral vasculature. Multiple chemotherapy agents and their adjuvant drugs are also associated with increased stroke risk, manifesting in different territories of the brain [45].

### 4.1. Direct Impact of the Tumour

The effect of the tumor itself in contributing to vascular events cannot be overlooked [43]. Stroke due to the direct impact of the tumor is relatively rare, albeit challenging to diagnose in clinical settings. Direct effects of tumor can manifest in varying forms [43], including compression of the blood vessel by tumor, invasion of arterial and venous sinus by leptomeningeal infiltrates or tumor mass, or bleeding within the tumor, also referred to as intratumoral hemorrhage (ITH) [46,47].

### 4.2. Compression

As the primary tumor grows or metastasizes, there is potential for accompanying edema to compress major intracranial blood vessels, such as when a lesion exerts a mass effect on a vessel or an opposing fixed cranial structure, such as the sphenoid bone or dural falx [38]. The impaired venous drainage and increase in capillary bed pressure can result in cerebral ischemia and infarction distal to the obstructed site [39]. This ischaemic stroke due to direct tumor compression is often seen in glioblastoma multiforme and brain metastases, impacting large vessels, in particular, the middle cerebral artery. Hemorrhagic stroke is comparatively less common, with the necrosis of highly vascularized tumor beds postulated as the mechanism behind this hemorrhagic conversion most commonly seen in melanoma, renal cell carcinoma, and choriocarcinoma [48].

### 4.3. Emboli

Stroke can occur from embolic phenomena when primary cancer metastasized to the heart, with melanoma, lung, breast, oesophageal, and hematological malignancies most likely to affect the heart [49].

### 4.4. Angioinvasive/Infiltrative

Hematological malignancies may also contribute toward stroke risk in cancer patients [50]. The hyper-viscosity associated with polycythemia vera, a type of blood cancer, can result in decreased perfusion of end vessels, leading to stroke. Furthermore, the infiltration of blood vessel walls in B-cell lymphomas was observed as manifesting in multiple vascular territory infarcts due to the infiltrative process of intravascular lymphomatosis [51]. Furthermore, dissemination of the tumor into the leptomeningeal space can congest vasculature and cause inflammation, resulting in downstream infarction [52].

### 4.5. Cancer-Mediated Hypercoagulability

In cancer-related stroke, beyond the conventional stroke mechanisms, cancer and its treatment may play a very important role in further accelerating stroke mechanisms, whether it be through creating a hypercoagulable state, accelerating the process of atherosclerosis, or through the adverse effects of therapeutic agents [41,43]. Abnormal coagulation cascades are an underreported yet important mechanism by which cancer can cause a stroke [41]. Coagulopathies such as disseminated intravascular coagulation are more likely noted in stroke patients with cancer than without [43]. Cancer-induced thromboembolic events originate from the molecular activity of cytokines, tissue factors, cancer procoagulants, and cancer mucin. The role of tissue factors and procoagulants accelerates the coagulation cascade, whilst inflammatory cytokines and cancer-derived mucin activate platelets. This hypercoagulable state associated with cancer is evidenced by the fact that in patients with cancer and stroke, most have elevated plasma levels of D-dimer, a non-specific marker of hypercoagulability [41]. D-dimers are a byproduct of fibrin clot degradation [53], thus serving as an unspecific yet sensitive measure of the degree of coagulation cascade activation and thrombus formation [13]. A prospective study of 140 stroke center patients with an additional active malignant cancer diagnosis identified D-dimer levels as being significantly higher in metastatic cancer patients, suggesting that cancer-mediated hypercoagulability is most prevalent amongst this group [41]. A study by Schwarzbach et al. indicated that specific DWI imaging patterns are involved in and characterize cancer-related stroke, involving lesions across multiple territories and elevated D-dimer levels—unique characteristics that may act to differentiate stroke vs. cancer-induced stroke [54].

### 4.6. Inflammatory Cytokines

Malignant cells attaching to vessel walls and adhering to the endothelium and extracellular matrix through membrane adhesion molecules can stimulate a local release of procoagulant cytokines, such as TNF-a, IL-1, and IL-6 [40]. These cytokines released by the tumor impact the underlying endothelium and lead to the sloughing of vascular endothelial cells and a phenomenon known as ‘blood sludging’ in which red blood cells congregate along blood vessel walls, reducing luminal diameter and rate of blood flow [55]. These inflammatory cytokines also induce endothelial cells, monocytes, and cancer cells to express tissue factor (TF), exerting a parallel action to potentiate the coagulation cascade. Protein C, which regulates Factor VIIIa and Factor Va (cofactors in the activation of Factor X and prothrombin), is inhibited by these cytokines [56]. Furthermore, thrombomodulin expression is downregulated, which normally works to bind to thrombin to inhibit coagulation activity [57]. Thus, the natural ‘brakes’ of the anticoagulation system are impacted. This cytokine storm was also implicated with higher rates of platelet activation and elevated levels of von Willebrand factor, which mediates platelet adherence to areas of endothelial damage [55].

### 4.7. Procoagulation Factors

Tumor cells release TF and cancer procoagulant (CP), both of which enhance the body’s existing inflammatory response. TF binds to Factor VII, potentiating the coagulation cascade by proteolytically activating Factor IX and Factor X, thus leading to the formation of thromboses and subsequent stroke [58]. The release of TF is in response to proinflammatory stimuli, such as the cytokines mentioned above of IL-1 and TNF-a released by tumor cells [55]. Elevated TF levels were observed in symptomatic atherosclerotic plaques in patients with carotid stenosis, thus suggesting that TF also destabilizes atherosclerotic plaques [38]. CP, a cysteine protease, is released in the majority of malignancies and directly cleaves Factor X → Xa, independent of FVII, resulting in thrombin generation, and 85% of cancer patients were shown to have elevated levels of CP, implicating its role in creating a procoagulant state for cancer patients [59].

### 4.8. Angiogenesis Inhibitors

In recent cancer therapy advances, tumor angiogenesis became a target, whereby a range of angiogenic factors, such as vascular endothelial growth factor (VEGF), insulin-like growth factor-1 (IGF-1), platelet-derived growth factor (PDGF), and epidermal growth factor (EGF), are inhibited [60]. The angiogenic network, however, is important in its role in preventing endothelial cell apoptosis, which can contribute toward atheroma development and complications, and coagulation activation in cancer patients. As such, anti-angiogenic-treated patients are more vulnerable to thrombotic events catalyzed by atherosclerotic plaque rupture and/or induced by the hypercoagulable state. Hypertension was also commonly noted with the use of angiogenesis inhibitors, with randomized controlled trials (RCTs) relaying a 2–3% incidence rate of hypertension and potential concurrent cardiac ischemia or infarction [61,62,63].

Antiangiogenic cancer drugs are mainly utilized in lung, kidney, colorectal, breast, gastrointestinal (GI), prostate, pancreatic, and hepatocarcinoma. These drugs are commonly sorafenib, and sunitinib, also linked to chemotherapy drugs, such as bevacizumab. Atherothrombotic events were noted in cancer patients treated with anti-angiogenic drugs with a median time of 7 months post-initiation of the drug, importantly underpinned by the drug-induced endothelial damage, which upregulates atheroma development and complications [64]. This is because the exposure of the drug-damaged subendothelium activates tissue factor and von Willebrand factor (vWF) to increase fibrin formation [65]. Furthermore, the hypertensive state created by anti-angiogenic drugs results in higher shear stress at sites of atherosclerotic plaques, thus increasing prothrombotic activity [66]. Antiangiogenic drugs also inhibit the anti-atherogenic actions of glucose uptake, lipogenesis, and anti-lipolysis which insulin accomplishes, leading to a hyperglycaemic and lipoprotein and free fatty acid (FFA)-rich environment, increasing atherothrombotic risk [66].

### 4.9. Cancer Mucin

Mucin is a potent procoagulant that directly activates prothrombin into thrombin, mostly secreted by adenocarcinomas [67]. Mucin is normally secreted by endothelial cells; however, adenocarcinomas, particularly of the pancreas, colon, breast, lung, prostate, and ovary, secrete this high-molecular-weight molecule directly into the bloodstream, potentiating a hyperviscous and hypercoagulable state [41]. Mucin also interacts with cell adhesion molecules on endothelial cells, platelets, and lymphocytes, forming platelet-rich microthrombi that deposit in vessels and lead to stroke [38]. *P*-selectin, a protein produced by activated platelets and endothelial cells to promote leukocyte adherence [68], interacts with carcinoma mucin to generate a platelet-rich microthrombi [40].

### 4.10. Non-bacterial Thrombotic Endocarditis (NBTE)

The exact prevalence of NBTE in cancer patients remains unknown, with the majority of the literature being case studies [42,69,70,71,72]. Sterile, thrombotic vegetations of platelets and fibrin can also deposit on cardiac valves (predominantly mitral and aortic), leading to arterial emboli, which may result in stroke [73]. NBTE is often associated with lung, pancreatic, and ovarian cancers and the aforementioned mucin-producing adenocarcinomas [55,74]. Cancer patients with NBTE typically present with widely distributed lesions in multiple territories, both large and small [75]. Approximately half of the patients with NBTE will present with thrombotic events such as stroke, with it often being the initial presentation, particularly in patients with late-stage pancreatic adenocarcinoma and non-small cell lung cancer [69,76]. This occurrence, of NBTE in cancer patients especially, is due to the damaged state of endothelial cells from the constant interaction between macrophages, monocytes, interleukins, TNF, and tissue factor, creating a surface for platelet aggregation, thus inducing a hypercoagulable state [42,71].

### 4.11. Neutrophil Extracellular Trap (NET) Formation

As part of the innate immune response, activated neutrophils release decondensed chromatin and form NETs, promoting platelet and coagulation factor activation and downstream thrombosis. By forming a scaffold for platelet, red blood cell, and fibrinogen adhesion, both the intrinsic and extrinsic coagulation pathways are activated. Patients with cancer-associated ischaemic stroke demonstrated increased NET formation levels, which in turn are linked to more traditional markers of coagulation and platelet activity, such as thrombin-antithrombin complexes and *P*-selectin levels [77]. Markers of NETosis include circulating deoxyribonucleic acid (DNA) released from neutrophils and citrullinated histone H2 [78]. A prospective study of 138 patients, of which 38 had cancer-related stroke, revealed plasma DNA and nucleosome levels to be statistically significantly elevated in cancer patients who had also experienced a stroke, with NETosis a likely coagulopathy mechanism [78].

### 4.12. Paradoxical Embolism and Patent Foramen Ovale (PFO)

Linked to NBTE, stroke via PFO and paradoxical embolism from deep venous thrombosis (DVT) are other important mechanisms to consider, seeing as one in four cancer patients have a PFO, and one in five had a venous thromboembolism (VTE) event [79,80]. Autopsy data also reveal that this rate may be as high as half of the cancer patients having a VTE [81]. A prospective study of 184 Japanese ischaemic stroke patients revealed that 18% had a right to left shunt (RLS), and 11% had a malignancy present [80]. This RLS was more frequent in cancer patients than in the ischaemic stroke—only cohort, at 55% and 15%, respectively. As such, we can gather that cancer patients are more likely to also have a PFO and those with DVT are at a higher risk of experiencing a paradoxical embolism. Thus, from a clinical perspective, testing for the presence of PFO should be pursued, and close monitoring for DVT is of utmost importance in the prevention of future stroke events. The interplay between these factors with the coagulation cascade is demonstrated in Figure 2.

## 5. Cancer Treatments and Stroke Risk

### 5.1. Radiotherapy and Stroke

Cancer patients on radiotherapy treatment were reported to be at an increased risk of atherosclerotic-related stroke, peripheral artery disease, and coronary artery disease [82]. Our understanding of the role of cancer within the picture of radiotherapy and atherosclerosis remains suboptimal, with hypotheses unanswered regarding whether cancer serves as a catalyst for atherosclerosis progression, or whether cancer itself is a trigger that instigates the process of atherosclerosis. There are a limited number of studies that investigate the role of radiotherapy in precipitating vascular events, with the most prominent summarized in Table 2.

The current understanding of the role of radiotherapy in increasing cancer patients’ stroke risk is that the chronic vascular injury and oxidative stress endured induces and accelerates the atherosclerotic process, particularly in the carotid artery, which often becomes stenosed [82]. There are two postulated theories as to how radiation therapy increases stroke risk within cancer patients: (1) by accelerating atherosclerosis through chronic inflammation; and (2) through a non-atherosclerotic picture of cerebral vasculopathy, known as moyamoya in severe cases [87].

In recent years, interest shifted to the damage that radiation causes to larger arteries such as the carotid. Previously, the exposure of the left anterior descending coronary artery to radiation in left-sided breast cancer patients warranted concern, and cohort studies show an increased cardiovascular risk in these patients [6,84,93,94]. The endothelial injury caused by radiotherapy, in particular to the carotid arteries, leads to a host of effects on the spectrum of initial atherosclerosis to occlusive atherosclerotic emboli presenting with stroke. Irradiated cells increase the production of reactive oxygen species, creating a picture of chronic inflammation, which increases the risk of atherosclerotic plaque rupture and plaque emboli lodging in cerebral vasculature [6,87,95]. It is thought that due to the necrosis and inflammation of endothelial tissue, the ensuing healing processes of proliferation and fibrosis stimulate and accelerate atherosclerosis, resulting in morphological features similar to that of spontaneous atherosclerosis [96]. Over time, this process of chronic inflammation increases the risk of atherosclerotic plaque and rupture [87].

A recent 2019 meta-analysis of 57,881 cancer patients found those receiving radiotherapy to be at a 2.09 times elevated risk of a subsequent stroke in comparison to the non-irradiated control groups [82]. Furthermore, within the cohort of irradiated radiotherapy, stroke risk was highest for the following groups: Hodgkin’s lymphoma (RR 2.81), head/neck/brain/nasopharyngeal cancer (RR 3.53), or if younger than 40 when treated or 40–49 years (RR 3.53 and 1.23, respectively). Interestingly, the geographical location of treatment also impacted patients’ risk of stroke, with the lowest risk in the US with a RR of 1.62, 1.88 in Asia, but a concerningly high RR of 4.11 in Europe. However, the authors noted potentially that this correlation may be confounded by patient factors, such as the fact that more terminal, sicker patients are given radiotherapy instead of chemotherapy [82]. A 2014 systematic review of 34 articles detailing the incidence of stroke/TIA in irradiated patients measured both carotid artery stenosis and carotid intima-medial thickening (CIMT) as surrogate endpoints to atherosclerosis. Nine studies were analyzed, of which, seven showed an increased stroke incidence in irradiated patients [6]. The RR ranged from 1.12 in patients with breast cancer to 5.6 in patients with head and neck cancer [92,97]. Overall, however, there are gaps in the existing literature; notably, lack of appropriate control groups, inability to account for confounding factors, and difficulties with long-term follow-up, resulting in conflicting literature as to whether radiotherapy increases stroke risk.

Most of these studies are retrospective in design, use a limited sample size, have a lack of appropriate control groups, have difficulties with long-term follow-up, and have an inability to account for confounding factors, with the predominant majority of patients identified as experiencing a vascular event with cardiovascular risk factors, notable diabetes mellitus, and dyslipidemia. As such, it is unclear as to whether the long-term risk after the initiation of radiotherapy identified stems from a cancer-based issue, a cardiovascular risk factor issue, or from the cancer treatment itself.

Although stroke risk induced by radiotherapy is established, the existing literature is conflicting and sparse when stratified into the specific cancer phenotype and radiation dose. Multiple limitations lower the generalisability of their findings, with these studies often retrospective in design, following a small sample size, and failing to account for the confounding cardiovascular risk factors of diabetes mellitus and dyslipidemia [85,87,88]. A small-scale prospective study of breast cancer patients was able to establish a control using the contralateral carotid artery, thus controlling for the influence of other atherosclerotic risk factors [98]. However, the limited sample size (*n* = 46) produced few results, with findings revealing no evidence of increased radiation-induced vascular injury or carotid intimal-medial thickening, contradicting other studies which found a significant relationship between radiotherapy and carotid artery stenosis and/or stroke risk [6,82,83,84].

A lack of studies with appropriate control groups and long-term follow-ups creates further gaps in the literature [6,84,86,89]. Studies by Dorresteijn et al. and Mueller et al., which followed patients for over a decade, identified the median time to stroke as being 10.9 years and 12 years from the initiation of radiotherapy, respectively [87,92]. These longer periods of time to the stroke event contrast with the majority of current literature, however, indicating a need for future studies that provide a long-term picture of patient progress in order to fully elucidate the continual stroke risk that cancer patients face. This is particularly pertinent for the cohort of childhood cancer patients who undergo radiotherapy, as the cumulative incidence of stroke in cancer survivors was shown to increase with age, with some stroke events occurring 30 years post-diagnosis [82]. Overall, this inconsistency in study design and findings point toward the numerous limitations of existing literature, with increased efforts in this field required to explore the true impact of radiotherapy on thrombotic risk, accounting for confounding factors, cancer phenotype, cohort demographic, and radiation regimen.

In summary, radiotherapy risk is inextricably linked with the process of atherosclerosis due to the inflammatory effect of irradiation, which promotes lipid plaque formation within damaged endothelium. As the majority of radiotherapy procedures are prescribed to those with locally targetable tumors, it emerges that the cancer phenotypes of head and neck, breast cancer, laryngeal and hypopharyngeal, and Hodgkin’s lymphoma are the most commonly studied and have the greatest wealth of studies conducted on the impact of radiotherapy on one’s stroke risk. Although the use of radiation therapy shows a declining trend across several pediatric cancer types [99], several studies indicated long-term stroke risk in children with cancer [92,100,101] and cancer survivors, with risks potentiating with longer follow-up, especially in those with childhood cranial tumors [102]. A variety of adverse effects arising from irradiation all contribute to this process of atherosclerotic emboli, resulting in a stroke. Several studies, beyond purely measuring the stroke risk, also quantified the degree of carotid artery stenosis, carotid intima-medial thickening, and calcification of the carotid artery, noting that these are all risk factors for future cerebrovascular events [101,103]. With regards to the relationship between radiation dose and stroke risk, it is hard to reliably quantify, given that most studies analyzed patients over several decades, during which radiation regimens underwent much change. However, general cumulative doses ranged from 27 Gy to 82.6 Gy, dependent completely on cancer phenotype, the severity of disease, and patient profile. The overall trend, however, is that higher daily fractions are associated with increased stroke risk, but that cumulative radiation dosage is just as important as longer radiation regimens, even at lower individual doses, which result in higher downstream stroke risk [104]. The timing of stroke post-radiation also differs depending on the patient cohort [105]. For adult cancer patients undergoing radiotherapy, the median time appears to be in the next few years (2–5) after the first radiation dose; however, for childhood cancer cohorts, the time to first stroke can be anywhere from 5 to 18 years [82,106].

### 5.2. Chemotherapy and Stroke

Previous studies indicated a putative risk of stroke in cancer patients receiving chemotherapy [44,107]. However, the exact level of this association and causative pathways remain poorly elucidated. Multiple chemotherapy agents and their adjuvant drugs are associated with an increased thrombotic risk, manifesting in stroke across different territories of the brain [45]. Certain chemotherapeutic agents, such as L-asparaginase (L-Asp), cisplatin-based treatments, 5-fluorouracil (5-FU), and bevacizumab (BVZ) were observed closely and implicated in thrombotic risk, with notable studies summarized in Table 3. Overall, thrombotic events for chemotherapy patients tend to involve not only cerebral vasculature, but also encompass VTE, potentially differing from patterns of radiotherapy-induced stroke due to their systemic effect as opposed to localized action near the cerebral circulation [43].

Whilst several systematic reviews and meta-analyses were performed to gauge the overall risk of stroke in cancer patients undergoing chemotherapy, research into specific drugs stratified by cancer phenotypes is required [117]. Research into a wider variety of chemotherapeutic drugs stratified by cancer phenotype and analysis of ischaemic vs. hemorrhagic stroke incidence is required to best guide clinicians in the administration of prophylactic therapy [117]. Our recent meta-analysis on VTE in cancer patients receiving chemotherapy revealed a pooled prevalence rate of 6%, ranging from 6% to 7% [118]. Furthermore, given the relatively increased risks of VTE in certain phenotypes of cancer, such as bladder, gastric, and ovarian, comprehensive cancer care should consider stratified VTE risk assessment based on cancer phenotype.

Albeit several studies into the effect of L-asparaginase on a cancer patient’s VTE/thrombotic risk were undertaken, there is a paucity of long-term studies that follow patients to track the long-term risk [109]. Furthermore, patient profiles often have confounding factors, such as concomitant use of steroids, anti-thrombin III supplementation, and type of steroid, none of which are properly stratified and deconstructed to adjust risk ratios [108]. However, other studies found vascular risk factors, such as hypertension, diabetes mellitus, and hypercholesterolemia to have no real variance across cancer patients who present with stroke, and those without [115].

In general, there is literature on stroke/vascular event risk, however, they detail purely case studies of singular events, and thus the overall thrombotic risk for wider populations is not elucidated [119]. With drugs such as fluorouracil and methotrexate, there is a lack of literature that has sufficiently large cohorts from which to draw conclusions. Furthermore, literature on methotrexate primarily details stroke-like neurotoxicity, which is not confirmed as a stroke on imaging. As such, there is more to be explored in this area concerning treatment for methotrexate-induced stroke mimics in particular, if traditional anticoagulants and antiplatelets have no efficacy.

The inability to account for confounding risk factors once again renders some study findings difficult to generalize given the impact of vascular risk factors, such as hypertension, diabetes mellitus, and hypercholesterolemia, in increasing the likelihood of stroke and/or VTE incidence [109,112]. However, other studies found vascular risk factors, such as hypertension, diabetes mellitus, and hypercholesterolemia, to have no real variance across cancer patients who present with stroke and those without, demonstrating a need for further research into potentially exacerbating risk factors [115]. Furthermore, the administration of concomitant immunomodulatory drugs and steroids was associated with a significant increase in thrombotic risk, with patients treated with thalidomide, doxorubicin, and dexamethasone shown to have a 10–27% increased VTE risk [120,121,122,123]. As such, the effect of adjuvant therapies on thrombotic risk must also be investigated and considered in future studies [108]. Several studies detail the effect of chemotherapy on VTE risk in the short term, identifying stroke risk as highest in the first few months following the first cycle of chemotherapy [109,110,116]. This means, however, that there is a paucity of long-term studies that follow patients to track the long-term risk of stroke once the chemotherapy regimen ended [109].

Certain chemotherapeutic agents, such as L-asparaginase, cisplatin-based treatments, fluorouracil, and bevacizumab, were observed closely in several studies, as they were implicated in increasing stroke risk [124]. For some patients, it is the adjuvant administration of dexamethasone and immunomodulatory drugs that further increase thrombotic risk and influence downstream stroke incidences. Overall, thrombosis in such patients does not involve cerebral vasculature, instead resulting in VTE events, potentially differing from radiotherapy due to their systemic effect as opposed to localized action near the cerebral circulation. Furthermore, for agents such as methotrexate, a stroke-like neurotoxicity is induced, as opposed to a definitive stroke, which can be confirmed upon imaging. Rates of thrombosis vary across pediatric and adult cohorts, and according to cancer phenotype and specific chemotherapeutic drugs [125]; however, the general trends are that older patients are at a much higher risk of VTE, longer periods of lower doses are associated with higher thrombotic incidence (dose-dependent effect), and most events occur during the induction phase of therapy—shortly after the first cycle of therapy is introduced to the body. Thrombotic risk is also more concerning for particular cohorts of patients undergoing chemotherapy [44,107], particularly those with renal cell cancer, colorectal cancer, and those in the advanced stages of cancer.

It would be of benefit to investigate more specifically the stroke risk associated with chemotherapy, distinct from pure VTEs. Furthermore, whilst L-asparaginase and cisplatin treatments are relatively well researched, the existing literature for stroke/thrombotic risk within patients receiving other chemotherapeutic drugs remains sparse, with case studies serving as the majority of evidence that such risk exists. The concentration of literature is on platinum-based therapies and L-asparaginase, but even between these drugs, quantifying ischaemic vs. hemorrhagic stroke incidence would help inform clinicians of treatment options.

Overall, more research needs to be conducted to provide a more comprehensive review of stroke and/or VTE risk across the breadth of chemotherapeutic drugs and their adjuvant therapies, again quantifying ischaemic vs. hemorrhagic stroke incidence in order to inform clinicians of the best treatment option.

#### 5.2.1. L-Asparaginase (L-Asp)

L-Asp is associated with an increased thrombotic risk-in particular, amongst cohorts of acute lymphoblastic leukemia (ALL) patients both pediatric and adult, as it is a common agent in the treatment regimen for ALL. L-Asp works by depleting the circulating levels of L-asparagine (ASN) and hydrolyzing ASN to L-aspartic acid and ammonia [126]. As ALL cells require ASN for protein synthesis and cell proliferation, a deficiency in ASN thus leads to apoptotic cell death of leukemia cells [127]. The mechanism by which L-Asp induces thrombotic events is primarily through its inhibition of hepatic coagulation factors, such as protein C, protein S, antithrombin, and fibrinogen, leading to a prothrombotic state [128].

#### 5.2.2. Cisplatin

Cisplatin is indicated in a range of cancers including bladder, head and neck, lung, ovarian, and testicular, operating by disrupting DNA repair mechanisms and inducing cancer cell apoptosis due to DNA damage [129]. Cisplatin-associated thrombosis was theorized as being a result of the direct endothelial damage incurred and the augmented procoagulant that cisplatin has, increasing tissue factor activity and von Willebrand Factor levels to foster a prothrombotic state. Furthermore, it was hypothesized that cisplatin reduces anticoagulant factor synthesis [111].

#### 5.2.3. Fluorouracil (5-FU)

5-FU is commonly indicated in the colon, head and neck, and breast cancers [130]. As an antimetabolite drug, 5-FU induces cytotoxicity by interfering with the action of thymidylate synthase (TS), an enzyme that catalyzes the conversion of deoxyuridine monophosphate to deoxythymidine monophosphate (dTMP); dTMP is a key metabolite in DNA replication and repair, and the inhibition of TS depletes dTMP, leading to double-stranded breaks in cancer cell DNA and an imbalance of intracellular nucleotides [131]. Additionally, 5-FU acts as a pyrimidine analog, misincorporating into DNA in place of uracil or thymine, ultimately causing cell death in rapidly proliferating cells [132].

Fluorouracil was identified as a risk factor for VTE in a Surveillance, Epidemiology, and End Results (SEER) database analysis of 11,086 patients with metastatic colorectal cancer. A single institution study found the rate of VTE to be 15% in patients receiving fluorouracil. When combined with granulocyte colony-stimulating factor (G-CSF), the incidence of VTE rose to 29% in metastatic colorectal cancer patients. This again, is conflicting, as in other randomized controlled trials, VTE rates with fluorouracil were reported in the low range of 1% [133].

The mechanism behind the VTE noted with 5-FU use can be explained by the acquired protein C deficiencies, which were noted particularly in breast cancer patients treated with cyclophosphamide and methotrexate in addition to fluorouracil [38]. Fibrinopeptide A and thrombin levels were also observed to alter with fluorouracil use. This may be in part exacerbated by the fluorouracil cardiotoxicity, which can underlie instances of thrombosis via direct myocardial toxicity, arterial vasoconstriction, endothelial damage, and changes in coagulation molecules.

#### 5.2.4. Immunomodulatory Drugs

Whilst not direct chemotherapeutic agents themselves, immunomodulating drugs (IMiDs), such as thalidomide and its derivatives of lenalidomide and pomalidomide, are administered adjuvant chemotherapy as combinatorial use to enhance anti-cancer immunity [45]. These IMiDs are most commonly used in multiple myeloma patients, as their mechanism of action consists of its tumoricidal effects of inducing cell cycle arrest within malignant cells, and anti-angiogenic properties [134]. Furthermore, IMiDs enhance the activity of T cells and natural killer T cells in secreting IL-2 and interferon-y, resulting in the inhibition of regulatory T cells, thus increasing myeloma-specific immunity [135]. The majority of literature published observes the impact of thalidomide and its derivatives.

As anti-angiogenic agents, thalidomide and lenalidomide are associated with a significant increase in stroke and venous thromboembolism (VTE) risk, particularly when used in conjunction with cytotoxic chemotherapy regimens and dexamethasone [45]. This is pertinent within the context of multiple myeloma, whereby the incidence of thrombosis on thalidomide alone is 5%, in comparison to a 10–20% risk when treated with thalidomide and dexamethasone, and for patients treated with thalidomide and chemotherapy, that risk increases to 20–40% [136,137]. The treatment regimen of thalidomide plus doxorubicin and dexamethasone was demonstrated to increase VTE risk by 10–27% at diagnosis, with a single study even reporting the risk to be 58% [120,121,122,123]. Similar results were observed with lenalidomide use, whereby VTE risk was highest in patients receiving high-dose dexamethasone, doxorubicin, or multiagent chemotherapy in conjunction with either thalidomide or lenalidomide [138]. It was noted with multiple myeloma also that the risk of VTE is higher in those patients who were newly diagnosed in comparison to relapses [138,139]. Interestingly, the chemotherapy drug bortezomib, however, was not shown to increase VTE risk in relapsed or refractory patients, and this may be of particular significance when considering the most appropriate therapy in treating recurring multiple myeloma patients [140].

## 6. Timing and Type of Stroke

Overall, amongst both cancer and non-cancer patients, the proportion of incidental ischaemic and non-ischaemic strokes remain consistent at 85% and 15%, respectively [141]. The highest risk of an ischemic stroke after cancer is within the first month of cancer diagnosis, particularly for those with stage 4 cancer, which is at a 10-fold increased risk compared to normal populations [142]. A population-based Swedish study from 2012 of 820,491 cancer patients hospitalized for a hemorrhagic or ischaemic stroke revealed the risk to be 2.2 and 1.6, respectively [143]. Though the overall stroke risk declined rapidly, it remained elevated at 1.2 (hemorrhagic and 1.1 (ischaemic) 10+ years post-cancer diagnosis.

In particular, for hemorrhagic stroke, the risk was particularly pronounced (greater than or equal to 2 times) in the first 6 months after diagnosis for 15 of the 34 cancers studied—importantly, that of the liver, small intestine, kidney, nervous system, thyroid and endocrine glands, myeloma, leukemia, and non-Hodgkin’s lymphoma. This risk in the first 6 months for all cancers averaged at 2.2, lowering quickly between 6 and 12 months to remain steady at 1.4 within 6–12 months, 1.3 after 1–5 years, and as aforementioned, 1.2 in 10+ years [143]. Concerning ischaemic stroke, the risk was increased in the first 6 months for 23 of the 34 cancers analyzed and was raised in the first 6 months post-diagnosis. Though it decreased, it also followed a steady trajectory, at 1.1 within 6–12 months, 1.1 after 1–5 years, and 1.1 after 10+ years [143]. This risk was more pronounced (greater than or equal to 2) in the first 6 months for cancers of the small intestine, pancreas, lung, nervous system, endocrine glands, and in leukemia.

Potential hypotheses for the increased risk of stroke within the first 6 months are: with continued treatment, tumor size reduces, and as such, associated inflammation and hemostatic activation also reduce; due to the death or successful treatment of cerebral metastasis patients who are most at risk, the risk will reduce along with the pool of the most vulnerable cancer patients decreases; smoking cessation soon after a cancer diagnosis, particularly in smoking-related cancers whereby tobacco is a risk factor for hemorrhagic or ischaemic stroke; and the initial psychological stress of a cancer diagnosis in the first 6 months and its interplay with cardiovascular disease [143].

For almost all cancer survivors, there is a temporal variation in the risk of having a stroke, which depends on the stroke phenotype and the time elapsed since the stroke. In a retrospective study, the incidence of recurrent stroke in cancer patients was found to be 31% within 3 months, including 13% with recurrent ischaemic stroke [144]. This is 3-fold higher than typical stroke recurrence in non-cancer patients [145]. Conversely, the risk of hemorrhagic stroke shows an upward trajectory with an increasing risk profile over 1 year [146].

Conversely, a point of interest is a diagnosis of cancer following an initial presentation with a stroke. A prospective study of 1282 patients with stroke for a mean of 27 months found 4.3% of patients to be diagnosed with cancer within 14 months of stroke, suggesting that for cryptogenic strokes, an occult malignancy can be the underlying cause [147,148]. This is indicative of the intertwined pathophysiology of stroke and cancer, whereby similar risk factors may predispose patients to both diseases, concurrently.

## 7. Discussion

In this paper, we provide a comprehensive update on the pathophysiology of stroke in cancer, an understanding of how stroke can severely impact cancer patients, and how cancer is associated with an increased risk of stroke. Cancer-related stroke, though underappreciated in clinical settings, may be a sizable proportion, warranting attention by clinicians as well as patients. Not enough attention is paid to cancer-related stroke as a subtype within the cryptogenic stroke, as demonstrated in Table 1, a sizable proportion, merits further investigation. Given the increasing prevalence of cancer, as well as the increased life expectancy of cancer patients, it is anticipated that there will be increasing cancer-related stroke risk in these patients, hence it is important for further awareness about this condition.

When observing the burden of stroke, cancer, and both diseases cumulatively, there is an unwarranted variation in the routine screening and clinical care of patients, with such procedures remaining suboptimal, particularly in LMICs [25,149]. Disparities within Australia most disadvantage the already at-risk and vulnerable populations of those who identify as Indigenous Australia, those living in regional and remote areas, and also those who have a lower socioeconomic status [150,151,152]. This is mirrored in the worldwide snapshot of stroke and cancer, whereby LMICs shoulder the majority of fatal stroke and cancer deaths and DALYs lost [153]. Bridging the gap between LMICs and the globe, and addressing the disproportionate burden of stroke, cancer, and stroke in cancer is also of utmost priority.

The importance of exploring stroke in cancer is evidenced (see Table 1) both by the burden of the diseases as separate entities and the burden created when the two diseases compound within a patient. As stroke and cancer share pathophysiological mechanisms, mapping these links is of utmost priority in enabling better management of cancer patients and contributing towards improved recovery profiles.

Currently, research into this area is lacking, with studies: failing to account for confounding factors such as dyslipidemia and diabetes mellitus, observing a limited sample size, and analyzing based on cancer phenotype with little comparison across the wider picture of cancer. Furthermore, beyond the research, limited clinical tools were developed to assist oncologists, neurologists, cardiologists, and general practitioners in identifying cancer patients with the highest risk of stroke. In a comprehensive 2019 population-based study of 7,529,481 cancer patients, the standardized mortality ratio (SMR) of a fatal stroke was identified as 2.17 [23]. The risk of fatal stroke was 21.64 per 100,000 person-years, and 80,513 (1.1%) died from a stroke. The patients who had a higher SMR included: patients diagnosed at an earlier age, and patients with metastatic disease. Overall, stroke risk among cancer patients was observed at more than twice that of the general population.

The pathogenesis of stroke in cancer patients is multifaceted, and often intertwines a combination of direct tumor effects of compression, tumor emboli, and infiltrative processes: cancer-induced hypercoagulability, the acceleration of atherosclerosis due to radiotherapy and resultant emboli, and chemotherapy-induced toxicity/damage [43]. The shared risk factors contributing toward the pathophysiology of cancer and stroke are pertinent in this context for practice and research, as a more thorough understanding of how they influence cancer patients’ stroke risk, with the confounding factors of atrial fibrillation, dyslipidemia, diabetes mellitus, and hypertension being most significant. Extensive research into the role of confounding factors in stroke risk is essential in order to isolate and definitively ascertain the stroke risk posed by chemotherapy and radiotherapy alone. Certain biomarkers of hypercoagulability, such as cancer mucin, TF, and CP, are potentially useful but non-specific in determining possible stroke risk [55,67]. By understanding the role of confounding vascular risk factors in contributing to stroke risk, measures can be taken to address these comorbidities in treatment too. As dose-dependent effects were noted, ascertaining dangerous levels of radiation and chemotherapy stratified by cancer phenotype, patient age, comorbidities, and time from diagnosis are important in informing therapeutic management. Understanding the difference in timing between ischaemic and hemorrhagic stroke occurrences post-diagnosis and post-treatment cycle will also play into this.

As stroke in cancer rates was not measured on a national or global scale, the burden of this was determined based on extrapolations of nationwide and global data available online. Due to the breadth of literature available on cancer-mediated hypercoagulability, only the most pertinent factors were covered. Furthermore, only a limited number of studies for each cancer phenotype are included in Table 2 and Table 3. As these papers themselves are already systematic reviews and meta-analyses, the summaries derived from these tables are generalized trends based on papers that already seek to capture data from a macro point of view. Furthermore, the outcomes measured across the studies collated are inconsistent within radiotherapy; both for stroke risk, carotid artery stenosis, and carotid intima-medial thickening, as well as atherosclerosis progression. Within chemotherapy, stroke risk is extended to vascular thrombotic events and stroke mimics. For chemotherapy-induced thrombotic risk (Table 3), only certain cancer phenotypes were explored as well, though there most certainly are other chemotherapeutic agents implicated. Though the role of steroids, such as dexamethasone, in increasing stroke risk is touched upon, they are not further elaborated upon/explored with studies. Finally, the role of antiplatelet therapy as a potential prophylactic avenue is lacking in the breadth of research.

The largest cohort study to date about the risk of stroke in cancer is by Zaorsky et al., utilizing data from the US Surveillance, Epidemiology and End Results (SEER) program on 7,529,481 cancer patients, which quantified the standardized mortality ratio to be 2.17 [23]. Whilst extremely comprehensive with regards to sample size, only the incidence of fatal stroke is reported at 1%, in comparison to other analyses, which quantify non-fatal stroke as well [23]. Furthermore, as the SEER database was utilized, information on stroke subtype, comorbidities, and the full extent of treatment was unable to be obtained, and thus, confounding factors were unable to be accounted for in the fatal stroke risk due to inconsistency in data collection. Furthermore, as patients were recruited from 1992 to 2015, treatment paradigms were adjusted; for example, Hodgkin’s lymphoma patients are now predominantly treated with chemotherapy as opposed to radiotherapy, thus conclusions about stroke risk within certain cancer phenotypes cannot be accurately drawn [154]. Finally, as patients who passed away in earlier years would have limited follow-up times and less time at risk, this may result in an overestimation of SMRs for those diagnosed in the latter years of the SEER data. This also applied conversely to those who were diagnosed in the most recent years, who would have shorter follow-ups and a lower chance of death, as well as benefitted from earlier detection and more advanced treatment, skewing the data.

In conclusion, stroke and cancer share important pathophysiological mechanisms. Deciphering these mechanisms is important to inform the optimal management of stroke or clotting risk in cancer.

## Figures and Tables

**Table 1:** Stroke in Cancer stratified by the burden of disease, prevalence, DALYs, and financial burden.

	Burden of Disease	Prevalence	DALY	Financial Burden
**Stroke**	**In Australia** [9]: 2.7% of the total burden of disease (2015) 38,000 incident cases (2017) 27,428 incident cases (2020) **Globally** [2]: 12.2 million incident cases (2019) 2nd leading cause of death, 11.6% of total deaths (2019) **LMICs** [8]: 70% of strokes and 87% of stroke-related deaths occur in LMICs (2017)	**In Australia:** 387,000 stroke survivors (2018) [10] 445,087 stroke survivors (2020) [3] **Globally** [2]: 101 million stroke survivors	**In Australia**: 4.6 DALYs per 1000 population (2015) [11] = 118,220 DALYs across the Australian population (2015) * 398.22 DALYs/100,000 population (2021) [12] = 102,342 DALYs across the Australian population (2021) * Globally [2]: 143 million DALYs, 5.7% of total DALYs (2019) **LMICs:** 87% of DALYs occur in LMICs [8]	**In Australia**: AUD 633 million was spent on health system expenditure (2015–16) [13] AUD 6.2 billion in direct financial costs (2020) [3] AUD 26 billion in lost well-being and premature mortality (2020) [3] **Globally**: Worldwide data N/R In the EU: EUR 27 billion in health system expenditure, EUR 1.3 billion in cost for informal care, EUR 12 billion in lost productivity (2017) [14] In the US: USD 156.8 billion in total cost, $103.5 billion in indirect costs, USD 38.1 billion from productivity loss, and USD 30.4 billion from premature death (2020) [15]
**Cancer**	**In Australia** [16]: 151,000 new diagnoses (2021) Estimated that 43% will be diagnosed with cancer by the age of 85 **Globally:** 23.6 million new cancer cases (17.2 excluding nonmelanoma skin cancer) (2019) [17]	**In Australia** [16]: 456,978 people living (who had been diagnosed with cancer) at the end of 2016 (diagnosed in 2012–2016) 1,176,285 people living (who had been diagnosed with cancer) at the end of 2016 (diagnosed from 1982 to 2016) **Globally** [17]: 85.8 million cases (2019) **LMICs:** 65% of all cancer deaths occurred in LMICs (2012), projected to be 75% in 2030 [18] Projection of 75% of the world’s cancers to occur in LMICs by 2040 [17]	**In Australia** [16]: 18% of total burden (2018) 881,094 DALYs (2018) **Globally** [17]: 250 million DALYs (2019)	**In Australia:** 9% of health system expenditure (2021) AUD 6.3 billion in yearly cost to the Australian healthcare system (for Australians diagnosed in 2009–13) (2013) [19] **Globally:** Worldwide data N/R In the EU: EUR 199 billion in total cost of cancer, EUR 103 billion on health expenditure on cancer care (including EUR 32 billion on cancer drugs), EUR 26 billion in informal care, EUR 70 billion in total productivity loss (EUR 50 billion from premature mortality, EUR 20 billion lost due to morbidity) (2018) [20] In the US: estimates of USD 161.2 billion on healthcare spending, USD 30.3 billion spent on productivity loss due to morbidity, USD 150.7 billion lost to premature mortality (2017) [21,22]
**Stroke in Cancer**	Country-specific data N/R A US population-based study of 7,529,481 patients found the risk of fatal stroke to be 21.64 per 100,000-person-years. Standardized mortality ratio is 2.17 (95% CI, 2.15–2.19) (2019) [23]	**In Australia**: Total number of cases of stroke in cancer (2019) = 0.06 × 456,978 = ~27,419 * Globally: The rate of non-fatal strokes in cancer patients is 5%, rate of fatal strokes in cancer patients is 1%. The combined rate of fatal plus non-fatal stroke is 6% for cancer cohort [23]. Total number of cases of stroke in cancer (2019) = 0.06 × 85.8 million = ~5.148 million *	**In Australia:** Total number of DALYs due to stroke in cancer (2011) = 0.06 × 833,250 = ~49,995 * Globally: Global DALYs due to stroke in cancer (2019) = 0.06 × 250 million = ~15 million *	**In Australia**: 0.06 × USD 6.3 billion of direct costs = ~USD 378 million of Australian health system expenditure on stroke in cancer (2013) *

Abbreviations: DALY: disability-adjusted life years, N/R: not reported, LMIC: low- and middle-income countries, EU: European Union, US: United States. * Extrapolated data, based on estimates of 2021 Australian and worldwide population numbers.

**Table 2:** Radiotherapy and atherosclerotic and thromboembolic outcomes in cancer patients.

Study	Cancer Phenotype	No. Controls vs. No. Patients	Incidence	Site of Therapy	Radiation Dose (Gy)	Interval from Radiotherapy (Months)	Overall Findings
Radiation-induced carotid artery atherosclerosis Gujral et al., 2014 [6]	Breast cancer Head and neck cancer Hodgkin’s lymphoma	Varied	*Stroke/TIA:* RR of stroke is 1.12 in patients with breast cancer. RR of stroke is 5.6 in head and neck cancer patients. *Carotid artery stenosis (CAS):* Increased prevalence of 16–55% in irradiated patients. *Carotid intima-medial thickness (CIMT):* Thickness increased in irradiated carotid arteries by 18–40%.	Varied	Varied	Varied	Measured a significant increase in stroke incidence in irradiated patients, and consistent differences in CAS and CIMT in irradiated and unirradiated carotid arteries.
Risk of ischaemic cerebrovascular events in head and neck cancer patients is associated with carotid artery radiation dose Van Aken et al., 2021 [83]	Head and neck cancer	*n* = 750 No control group.	A total of 27 patients (3.6%) experienced an ischaemic cerebrovascular event (ICVE). The 5-year cumulative risk of stroke is 4.6%. The 8-year cumulative risk of stroke is 7.4%.	Solid tumours located in the larynx (45%) and the oropharynx (36%).	Mean dose to carotid arteries = 39.8 ± 0.5 Gy.	The mean time to event is 22.8 months. Mean age at ICVE = 63 years.	The increased risk is associated with an increased dose to carotid arteries. Maximum dose significantly associated with ICVE risk. The absolute volume of the carotid arteries that received at least a radiation dose of 10 Gy is most important prognostic factor.
Radiation to supraclavicular and internal mammary lymph nodes in breast cancer increases the risk of stroke Nilsson et al., 2009 [84]	Breast cancer	Nested case-control study of a cohort of 4689 women with invasive breast cancer. *n* = 282 women diagnosed with breast cancer, hospitalized for stroke. Control group = 282 women with breast cancer.	282 cases of stroke = 6% of cohort. 10% of cases were hemorrhagic stroke, 2% subarachnoidal, and 8% intracerebral. 62% of cases had ischaemic stroke, 48% were infarction, 14% had transient cerebral ischemia. 80% of strokes originated in carotid arteries, 20% from the vertebrobasilar system.	Combination of the thoracic wall, internal mammary chain, supraclavicular, and axillary lymph nodes.	Different RT regimens across 1970–2003, varying from 20 Gy to 54 Gy.	Not specified	Radiotherapy to the internal mammary chain and supraclavicular lymph nodes was associated with a higher risk of stroke compared to no radiotherapy, OR = 1.3 (95% CI, 1.1–2.8), however not statistically significant. Statistically significant trend for increased risk of stroke with higher daily fraction dose.
Vascular events from carotid artery atherosclerosis after radiation therapy for laryngeal and hypopharyngeal cancer: the incidence and risk factors Makita et al., 2020 [85]	Laryngeal and hypopharyngeal cancer	*n* = 111 patients (95 laryngeal, 16 hypopharyngeal) No control group.	5.4% (6 of 111 patients) The 5-year occurrence rate is 5.5% (95% CI, 0–10.5%). The 8-year occurrence rate is 10.7% (95% CI, 1.4–19.1%).	For laryngeal cancer and dependent on spread of disease-vocal cords, 1 cm margin surrounding the tumour For hypopharyngeal cancer–gross tumor plus 1 cm margin, prophylactic lymph node areas.	Median of 66 Gy. Range of 60–74 Gy.	Median occurred at 51.7 months. Range of 0.3–78.3 months after radiotherapy initiation.	Dyslipidaemia, diabetes mellitus and carotid calcification are important factors for event occurrence. As 3 of 6 cases occurred out of the field of irradiation, no carotid artery parameters were significantly correlated with a vascular event.
Changes in the Common Carotid Artery after Radiotherapy: Wall Thickness, Calcification, and Atherosclerosis Kim et al., 2017 [86]	Laryngeal cancer patients	*n* = 125 No control group.	Calcification in 37 patients (29.6%) and atherosclerosis in 71 patients (56.8%).	Primary tumor site, neck region, entire common carotid artery area.	Cumulative radiation dose ranged from 27 to 82.6 Gy.	Mean of 62.7 ± 32.1 months after radiotherapy for radiation-induced atherosclerotic changes.	Atherosclerosis in the middle portion of the common carotid artery occurred in 24.6% of patients, at the proximal CCA at the intrathoracic level in 20.6% of patients, and at the distal CCA in 4.8% of patients. Demographic, risk factors and radiation doses were not found to be associated with the change in CCA wall thickness.
Risk of First and Recurrent Stroke in Childhood Cancer Survivors Treated with Cranial and Cervical Radiation Therapy Mueller et al., 2013 [87]	Childhood cancer	*n* = 325 No control group.	A total of 19 first strokes occurred over the period of the study from 1980–2009. 13 ischaemic, 4 haemorrhagic, 2 unknown. The cumulative incidence of the first stroke is 2% at 5 years after irradiation, 4% at 10 years after irradiation. A total of 6 recurrent strokes. The median time to recurrence is 15 months after the first stroke; 38% at 5 years, 59% at 10 years	Cranial and cervical radiotherapy	Range from 29.5 Gy–72 Gy.	The median time to develop the first stroke after RT was 12 years (144 months), IQR is 5–18 years. Recurrent stroke occurred with a median time of 15 months.	Each additional year of age at the time of radiation increased stroke risk by 12%. With each 100-cGy increase in radiation dose, stroke hazard increase by 5%.
Radiation, Atherosclerotic Risk Factors, and Stroke Risk in Survivors of Pediatric Cancer: A Report from the Childhood Cancer Survivor Study Mueller et al., 2013 [88]	Childhood cancer	Retrospective cohort study *n* = 14,358 five-year survivors of childhood cancer Control group = 4023 random selection sibling controls.	A total of 292 survivors reported late-occurring stroke, 23.3 years mean follow-up. Stroke rate of 77 per 100,000 person-years, 9.3 in siblings RR is 7.8 (95% CI, 4.7–13.0) compared to control siblings.	Varied	Varied Range from 0–50 + Gy	Time after radiotherapy unable to be found The mean time from diagnosis to late-occurring stroke was 18.6 years Range time was 5.2–38.1 years Cumulative incidence in survivors treated with 50+ Gy CRT is 1.1% at 10 years post-diagnosis and 12% at 30 years post-diagnosis. The median age of stroke was at 29 years of age.	Dose-dependent hazard ratio, 5.9 for 30–49 Gy CRT, 11.0 for 50+ Gy CRT. Among young adult survivors with 50+ Gy CRT, 12fold cumulative incidence of stroke 10–30 years post-diagnosis.
Radiotherapy Exposure in Cancer Patients and Subsequent Risk of Stroke: A Systematic Review and Meta-Analysis Huang et al., 2019 [82]	Cancer survivors	From 12 eligible studies, *n* = 57,881 Control groups existed of cancer patients receiving non-radiotherapy treatments.	RR 2.09 (95% CI, 1.45–3.16) in radiotherapy vs. no radiotherapy cancer patients.	Varied	13–80 Gy	N/A	Overall doubling of stroke risk in cancer patients.
Effects of Neck Radiation Therapy on Extra-Cranial Carotid Arteries Atherosclerosis Disease Prevalence: Systematic Review and a Meta-Analysis Bashar et al., 2014 [89]	Malignant head and neck tumors - squamous cell carcinoma, nasopharyngeal carcinoma, etc.	Combination of 8 studies, 1070 patients total *n* = 596 Control group = 474 non-irradiated cancer patients.	Risk ratio is 4.38 for overall stenosis, 7.51 for severe stenosis. *Abnormal scan:* 6 studies, 908 patients total; 237/534 of patients in the RT group had some degree of stenosis, vs. 33/374 in the control group *High-grade stenosis:* 5 studies, 717 patients. >70% stenosis in 51/406 of the RT group vs. 3/311 of controls. *Low-grade stenosis:* 5 studies, 770 patients; 89/454 in RT group, vs. 21/316 in the control group.	Head and neck	Variable as protocols differ across institutions and diseases.	N/A	Extracranial carotid artery stenosis is higher in patients receiving radiotherapy for neck malignancies. Progressive thickening of intima-media early in the first 12 months following radiotherapy initiation. Acceleration of the process of thickening is estimated to be 21 times higher than control groups undergoing no radiotherapy [90].
Predictors of carotid artery stenosis after radiotherapy for head and neck cancers Chang et al., 2009 [91]	Head and neck cancer	*n* = 192 Control group = 98 patients not undergoing radiotherapy.	Carotid plaque score significantly higher in the irradiated group, *p* < 0.001.	Whole pharynx, skull base, whole neck lymphatic system.	All upper neck areas at least 6000 cGy of radiation 18–20 Gy daily fraction, 5 fractions a week. The total median value of 7060 cGy to the initial area of gross disease.	Time intervals only available for the RT group The average time after RT is 2 years to develop carotid artery stenosis.	Bilateral plaque score for carotid artery stenosis is significantly correlated with age, hyperlipidemia and radiotherapy.
Increased risk of ischemic stroke after radiotherapy on the neck in patients younger than 60 years Dorresteijn et al., 2002 [92]	Head and neck cancer	*n* = 367 patients No control group.	In 14 cases of stroke, RR is 5.6 (95% CI, 3.1–9.4), and 15-year cumulative stroke risk is 12%.	Varied	50–66 Gy	Mean of 10.9 years.	Analysis of risk factors revealed hypertension and DM to increase RR after RT. After more than 10 years of follow-up, RR elevated to 10.1 (95% CI, 4.4–20).

**Table 3:** Chemotherapy regimens and stroke outcomes in cancer patients.

Study	Cancer Phenotype	Therapeutic Drug	Cohort Size	Stroke Incidence	Impact/Other Findings
Caruso et al., 2006 [108]	Children with acute lymphoblastic leukemia (ALL)	L-asparaginase/anthracyclines/prednisone	*n* = 1752 children from 17 prospective studies No control group.	Rate of thrombosis 5.2% (95% CI 4.2–6.4)	Most events occurred during the induction phase of therapy. Lower doses for longer periods are associated with the highest thrombotic incidence.
Grace et al., 2010 [109]	Pediatric and Adult patients with ALL	L-asparaginase	*n* = 548 patients No control group.	8% experienced VTE 5% of pediatric and 34% of adult patients	Timing: for 8 patients, VTE occurred during induction, post-induction in 35 patients Median time to VTE was 3.5 months from initiation of therapy (range 0.5 to 10.1 months) Age is a significant predictor of risk, >30 years deemed very high risk with a VTE rate of 42%
Li et al., 2006 [110]	Various	Various	*n* = 10,963 patients with malignancies followed up at 1-month post chemotherapy No control group.	15 patients experienced 16 ischaemic strokes within first month after the latest chemotherapy Incidence of a post-chemotherapy stroke at 0.137%.	Drug type: Cisplatin was administered prior to 8 ischaemic strokes, oxaliplatin to one. 75% occurred within 10 days of the latest chemotherapy, 10 happened after first cycle of chemotherapy.
Zahir et al., 2017 [111]	Various	Cisplatin	*n* = 200 patients receiving cisplatin Control group = 200 patients on non-cisplatin-based regimens	31 VTE events in the cisplatin group, the cumulative dose was 471 mg/m^2^ Group without events had a mean cumulative dose of 322 mg/m^2^ The crude RR of VTE in the cisplatin group is 2.8 (95% CI, 1.4–4.2) compared to the non-cisplatin group Adjusted for gender, ECOG, and presence of central venous catheter, the adjusted RR is 3.32.	Baseline characteristics of diabetes mellitus, hypertension and coronary artery disease were similar across both cisplatin and non-cisplatin groups. High incidence of VTE in patients receiving cisplatin-based chemotherapy.
Seng et al., 2012 [112]	Various	Cisplatin	*n* = 8216 patients from 38 RCTs No control group.	A 1.92% incidence of VTEs in cisplatin-receiving patients vs. 0.79% incidence in non-cisplatin-based regimens. RR of 1.67 (95% CI, 1.25–2.23) of VTE for the cisplatin-receiving group	Dose-dependent effect noted. Cisplatin is associated with a significant increase in VTE risk in patients with advanced solid tumours.
Hoy et al., 2009 [113]	Early breast cancer	5-fluorouracil, epirubicin, cyclophosphamide (FEC)	*n* = s 176 patients receiving FEC regimen Control group = 149 patients receiving other chemotherapy regimens.	27% incidence in FEC receiving group (47/176), with 5% of patients on other regiments experiencing VTE (7/149).	Adjuvant FEC chemotherapy is associated with increased VTE incidence for patients with early breast cancer.
Watanabe et al., 2018 [114]	Leukaemia or lymphoma	Methotrexate (MTX)	*n* = 9 patients No control group.	Of 44.4% (4/9) of patients with leukaemia/lymphoma and episodes of stroke-like presentation were diagnosed with MTX-induced stroke-like neurotoxicity, 22.2% (2/9) had disturbed consciousness, speech disorders, hemiparalysis.	MTX-induced neurotoxicity may manifest in a stroke-like presentation, difficult to distinguish from stroke. Neurological events occurred 10–13 days after the 4th or later MTX treatment.
Auer et al., 2017 [115]	Recurrent glioblastoma multiforme (GBM)-1st/2nd/3rd relapse	Bevacizumab (BVZ)	*n* = 40 treated with BVZ (178 scans) Control group = 42 patients matched for age and gender receiving basic treatment (186 scans).	An 8% (7/82) incidence of vascular events from MRI scans, with 4 events recorded in BVZ group, 3 ischaemic stroke, and 1 intracranial haemorrhage; 3 events in the control group 1 ischaemic stroke, and 2 intracranial haemorrhages.	BVZ treatment not associated with increased risk for vascular events in recurrent GBM patients.
Ranpura et al., 2009 [116]	Variety of solid tumours	Bevacizumab	*n* = 12,617 patients from 20 RCTs No control group.	The incidence of all-grade arterial thromboembolic events was 3.3% (95% CI, 2.0–5.6). The incidence of high-grade ATE is 2.0% (95% CI, 1.7–2.5). With BVZ, RR of 1.44 compared to controls of having an arterial thromboembolic event.	The risk did not increase with BVZ doses, with 2.5 mg and 5 mg/kg/week having an RR of 1.52 (95% CI, 1.10–2.09) and 1.50 (95% CI, 0.84–2.69), respectively. Increased risks for patients with renal cell cancer (RR 3.71) and colorectal cancer (RR 1.89). High-grade cardiac ischemia is higher than controls at RR 2.14, but the risk of ischaemic stroke is not significantly different from controls with an RR 1.37. The median time to the first event is 2.6 months in BVZ treated group, vs. 2.1 months in the control group.

## Data Availability

The original contributions presented in the study are included in the article, and further inquiries can be directed to the corresponding author.
